# Developing and Applying a Usability Testing Methodology for a Website With Survivors of Stroke and Caregivers: Tutorial

**DOI:** 10.2196/86143

**Published:** 2026-08-18

**Authors:** Katie Nesbitt, Niranjan Bidargaddi, Richard Cullen, Erin Godecke, Coralie English, Dominique A Cadilhac, Adrian O'Malley, Annette McGrath, Elizabeth A Lynch

**Affiliations:** 1 College of Health and Enablement Caring Futures Institute Flinders University Adelaide, South Australia Australia; 2 National Stroke Foundation Melbourne Australia; 3 Stroke Recovery and Rehabilitation Perron Institute for Neurological and Translational Science Perth Australia; 4 Allied Health Research Sir Charles Gairdner Osborne Park Health Care Group Perth, Western Australia Australia; 5 School of Health Sciences University of Newcastle Australia Newcastle Australia; 6 Hunter Medical Research Institute Newcastle, New South Wales Australia; 7 Centre for Research Excellence to Accelerate Stroke Trial Innovation and Translation Sydney Australia; 8 Stroke and Ageing Research Department of Medicine Monash University Melbourne Australia; 9 Stroke and Critical Care Public Health and Health Services Research Florey Institute of Neuroscience and Mental Health Melbourne, Victoria Australia; 10 Person with lived experience of stroke Sydney Australia; 11 Person with lived experience of stroke Adelaide Australia

**Keywords:** usability testing, stroke survivors, accessibility, websites, caregiver

## Abstract

Survivors of stroke, particularly those with communication difficulties, are often excluded from usability testing of digital health tools. This exclusion limits the accessibility and relevance of digital interventions to this population. This tutorial presents a tailored methodology for usability testing of a co-designed website, EmpowerMe, developed to enhance self-efficacy in survivors of stroke and caregivers. A multidisciplinary team created a usability conceptual framework that included survivors with and without communication difficulties. Testing procedures were adapted to accommodate cognitive, motor, and communication needs. Outcome measures included usability scores, task completion rates, knowledge acquisition, and user experience feedback. The tutorial offers a replicable framework for inclusive usability testing in digital health, especially for populations with neurological and communication impairments.

## Introduction

Usability testing is a critical component of digital design, encompassing learnability, efficiency, memorability, error prevention, and user satisfaction [1,2]. Stroke is a leading cause of disability worldwide, often resulting in cognitive, physical, and communication impairments [3]. The diverse range of poststroke disability highlights the importance of rigorous usability testing to ensure that interventions are both accessible to and effective for survivors of stroke. Despite its importance, survivors of stroke, particularly those with communication difficulties, are frequently excluded from formal usability testing, creating inequitable and inaccessible digital health designs and limiting applicability [4-7]. This exclusion of survivors of stroke with communication difficulties such as aphasia (a communication disability affecting participation and access to information) and apraxia of speech (difficulty accessing motor pathways required for speech) has created an inequitable evidence base in stroke research, often necessitating repeated studies with survivors of stroke with communication difficulties [8,9].

Worldwide, stroke is the second leading cause of death, accounting for 11.6% of all deaths [10]. Stroke can be devastating to survivors and their families when they suddenly acquire a permanent disability for which they are completely unprepared. Successful management of long-term health conditions relies on self-efficacy, the belief in one’s ability to perform behaviors necessary for achieving specific goals, and self-efficacy is also a critical factor in stroke recovery [11]. It supports engagement in rehabilitation, fosters independence, and helps individuals navigate the complex emotional and physical challenges following a stroke. Higher self-efficacy is associated with less depression and better quality of life and a greater physical ability to move around and execute tasks [11]. However, survivors of stroke are rarely given advice about programs that could enhance their self-efficacy [12], suggesting that self-efficacy is poorly addressed in this population. Our team developed a website, EmpowerMe, through collaboration with people with lived experience of stroke (a working group) and the Stroke Foundation (Australia) to build self-efficacy in survivors (Australian New Zealand Clinical Trials Registry number 12624001018505) [13].

During website co-design work group meetings, the lived experience working group comprising survivors of stroke and carers emphasized the importance of being able to find and understand information that was relevant to them. This underpinned the ongoing design sessions, which focused on workshopping features such as intuitive navigation, tailored content, and presentation formats that optimize learning and retention. Survivors and carers prioritized the need to enhance self-efficacy for individuals coping with communication or cognitive changes, fatigue, or physical disability. Their insights shaped not only the design of the EmpowerMe website but also the development of a usability testing methodology that could accommodate diverse needs.

To address the gap in inclusive usability testing, this tutorial describes the development and application of a tailored methodology that we used with survivors of stroke with and without communication difficulties and caregivers. Our goal is to provide a practical framework for researchers and designers working with neurologically diverse populations.

### Design of Usability Testing Procedures

One methodology available to conduct usability testing is a test series approach, which involves multiple structured sessions designed to evaluate the usability of a product or service across key domains such as ease of use, efficacy, efficiency, accessibility, and user satisfaction [14]. This approach was selected for testing the EmpowerMe website as it allowed for iterative refinement and in-depth observation of user interactions.

Usability tests are research activities that rely on the direct involvement of a product’s intended users. In this methodology, survivors of stroke and caregivers were central to the evaluation process. Their interactions with the website were observed synchronously (sessions and data collection were conducted in real time) either online or in person, and their feedback was collected through posttask interview questions. This dual approach enabled the research team to identify usability barriers and opportunities for improvement.

### Ethical Considerations

This project received ethics approval (5886) from Flinders University’s Human Research Ethics Committee. Usability testing was conducted in accordance with established ethical standards to ensure the integrity of the study and the protection of all participants and data involved. Informed consent was obtained prior to participation, and individuals were free to withdraw at any time. The research speech pathologist (EG) prepared an aphasia-friendly participant information sheet and explained the information to each participant with aphasia using aphasia-friendly language and allowing time for processing of information. She checked the participants’ understanding of the information as part of the consent process. Confidentiality and privacy were maintained through secure data handling practices, with deidentification of collected data and only the research team having access to them.

### Conceptual Framework

The conceptual framework guiding this usability methodology outlines the full cycle of testing activities, from initial preparation to final analysis. This framework comprises a series of steps with corresponding considerations and case examples from our testing (Table 1). It was designed to ensure that each stage of the process could be adapted to meet the needs of survivors of stroke, including those with communication difficulties. These include determining testing objectives, identifying and recruiting users, tailoring test procedures, pilot-testing procedures, and conducting testing and analysis. The framework was generated through discussions with the multidisciplinary research team, which included digital designers, health professionals, survivors of stroke, and carers. Usability testing strategies were informed by discussions with the lived experience work group. Central to our framework was the integration of lived experience. Survivors and caregivers from the lived experience working group helped identify barriers to digital access and shaped the testing procedures so that the intended users (individuals with varying consequences of stroke) could be recruited and appropriately supported as usability testing participants. Throughout each phase, specific adaptations were made to support participation by individuals with varied poststroke challenges, including communication difficulties. These included the use of aphasia-friendly language, teach-back techniques, and personalized technology setup support. The framework served not only as a planning tool but also as a guide for responsive, inclusive testing practice.

**Table 1:** Usability testing conceptual framework for survivors of stroke.

	Considerations	Case example of the planning process for this project
Step 1: determine testing objectives	How are you anticipating users will interact with your website?	We sought to test the following: (1) can people navigate through the website and find relevant information? (2) Does the content make sense to the users?
Step 2: identify and recruit users	Who are the intended users? Are there intended users who might have more difficulty using the website?	We recruited three cohorts: (1) survivors of stroke without communication difficulties, (2) survivors of stroke with communication difficulties, and (3) carers
Step 3: tailor test procedures	What barriers might the intended users face when accessing the website?	Survivors of stroke and carers advised that survivors might have difficulty using online resources due to (1) fatigue; (2) communication difficulties; (3) visual changes, including visual loss and visual inattention; (4) motor changes; (5) sensory overload; and (6) distractability
Step 4: pilot-testing procedures	How can identified barriers from the previous step be addressed?	Suggested strategies to incorporate into testing: (1) a quiet location; (2) allowing participation from home (especially if the person uses adaptive equipment); (3) 1:1 moderation; (4) allowing for rests; (5) time-limited sessions; (6) extra time for technology setup; (7) communication difficulty–friendly consent processes; and (8) communication difficulty–friendly testing procedures, including in-person testing
Step 5: testing and analysis	How will the testing be conducted? What tools are required to collect data? What data can be collected to measure how users interact with the website? What data are feasible to collect given the needs of participant groups? How should the data be analyzed?	In-person or online synchronous testing, usability scripts (delivered in person for people with communication difficulties by an expert in aphasia-friendly principles), SUS^a^ (usability), experience survey (we measured time taken to complete tasks, percentage of tasks completed, usability, and user experience—including suggestions for improvement), descriptive statistical analyses (time and completion, knowledge testing, and survey) and *t* tests (SUS scores), and SUS Calculator software package [15]

^a^SUS: System Usability Scale.

### Steps

#### Step 1: Determine Testing Objectives

##### Considerations

In any usability test, the design team should consider how they anticipate users will interact with the digital resource and test these assumptions. Typically, usability testing will evaluate navigation and technical features of the resource, along with appropriateness of content for the intended user group. Our usability testing focused on 4 core areas: website functionality, navigation and ease of use, relevance and clarity of information, and overall user experience.

##### Case Example

**Figure 1:**
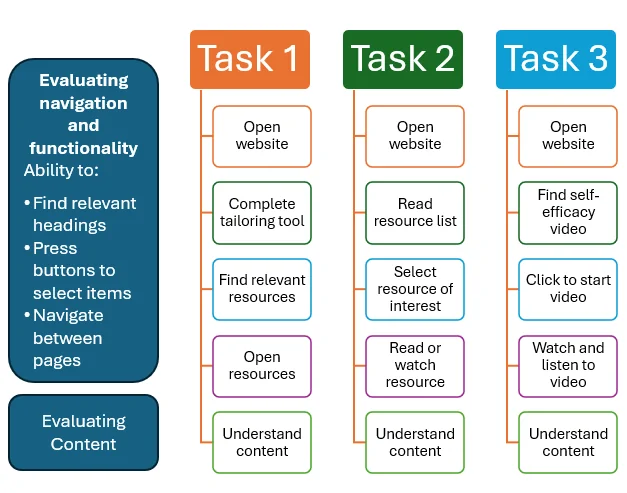
Usability tasks and anticipated actions.

During the website build, features were incorporated into the website to enhance tailoring of content to individuals’ needs and interests. The design team developed assumptions about how users would interact with the website and how they would find information (Figure 1). The anticipated actions of website use were watching the landing video, navigating to and completing the tailoring tool (a questionnaire that centralizes content within the website that is relevant to the user), navigating to tailored resources, navigating to and proceeding to watch self-efficacy videos (practice, encouragement from others, skill development, learning from others, and information), navigating between website pages and tasks, and navigating back to the EmpowerMe website when a resource has opened in a new window.

#### Step 2: Identify and Recruit Intended Users

##### Considerations

Digital health interventions should be tested by users who reflect the target group. It is important to identify any subgroups who might interact with the resource differently or face challenges using it and ensure that they are included in usability testing.

##### Case Example

The intended users of the EmpowerMe website were survivors of stroke and their caregivers. Three discrete groups of users were identified to include in usability testing: (1) survivors of stroke with communication difficulties; the team considered that, if the website were accessible to people with communication difficulties, it would also meet the accessibility needs of people with mild cognitive changes; (2) survivors of stroke without communication difficulties; we also wanted to include survivors of stroke without communication difficulties to see whether the structures and content were pitched at the right level for people without communication impairments but potentially living with other challenges such as visual changes or fatigue; and (3) caregivers; we sought to include caregivers because more than 50% of survivors of stroke living in the community require family caregiver assistance [16,17], and carers of survivors of stroke frequently play an important role in finding relevant information and making health care decisions after the stroke, as well as supporting the survivor in building their self-efficacy.

Users were recruited through advertising on the Stroke Foundation (Australia) EnableMe website and newsletters and through invitation emails sent to distribution lists associated with the project. An online booking page was linked to the project team’s calendar, and prospective users could book their own session or book directly through the project manager if preferred. When the booking was confirmed, prospective users were emailed an information sheet, a consent form for signing, and instructions about what to expect. Users who would complete an online usability testing session were sent instructions on how to use Zoom (Zoom Communications, Inc). The research speech pathologist explained the information sheet to each user with communication difficulties using aphasia-friendly language and allowing time for processing of information. Participants’ understanding of the information was checked as part of the consent process.

#### Step 3: Tailor Testing Procedures to the Needs of the Intended Users

##### Considerations

When planning usability testing, it is important to consider potential barriers that the intended users may face when accessing the digital resource. Testing procedures should evaluate whether the barriers have been suitably addressed during the design, and strategies should be used that account for these barriers during testing (eg, simple instructions for people with cognitive changes and supported communication using aphasia-friendly resources for people with communication difficulties). Expertise or specialist training are maybe required to address accessibility needs. Factors to consider in all usability testing include digital literacy, vision, dexterity, and how concentration levels could impede a person’s ability to interact with the features of the digital resource. Other factors such as age of the intended users, literacy and educational levels, and language-related factors that affect how users engage with the content should also be considered. Finally, the space in which testing will be conducted, how long the procedures will take, the complexity of the testing activities, and how these influence the needs of the intended users should be considered.

##### Case Example

We considered common challenges arising from strokes, such as communication difficulties that result in individuals having difficulty understanding verbal instructions or communicating their responses; cognitive or attention changes; visual field loss; and neurological fatigue, which is often exacerbated by looking at screens and when concentrating [18]. To avoid distraction and sensory overload, usability testing for all participants was conducted in a one-to-one moderated format rather than in a group.

The lived experience working group recommended online usability testing for carers and for survivors of stroke without communication difficulties so that they could participate without the need to travel, which can contribute to fatigue and stress when the venue is at an unfamiliar location. Furthermore, participating in usability testing from their homes would allow survivors to use adaptive equipment if required. After a stroke, adaptive equipment can support users through both hardware and software solutions. Some examples of these are sticky keys, voice recognition help with typing and keyboard shortcuts, large key or one-handed keyboards, and eye-tracking systems. These adaptations could not be accommodated for in-person testing as they are often hardware aids fitted to personal devices. We needed to record the session on our software systems. Sessions were limited to 75 minutes to avoid fatigue, which allowed for 15 minutes for setup of technology and equipment and 60 minutes for usability tasks. Having the option to participate in person was also suggested if the survivor of stroke or carer preferred not to participate online.

To meet the needs of people with communication difficulties, in-person usability testing was planned with an aphasia-friendly delivery of the script. These sessions were comoderated by a speech pathologist with expertise in working with people with communication difficulties. Three hours were allocated for the sessions to allow for regular breaks and extended time to complete tasks to avoid undue fatigue within sessions.

#### Step 4: Pilot-Testing Procedures

##### Considerations

Pilot-testing procedures run through a usability session in advance of the planned testing. This usually involves 1 to 2 sessions and is important to validate the wording of the script, determine how long the session will take, and highlight any other issues that could compromise the effectiveness of the session [19]. It can help find problems, including whether the participants understand the instructions and tasks, whether the tasks elicit the required information to address the assumption, whether the tasks can be completed, and the time required to complete the tasks [19].

##### Case Example

The online usability testing procedures for this project were pilot-tested with 2 test users to identify any human or system issues. Sessions required additional time to allow for users’ technology skills and aptitude. There were system delays at the start in the form of Zoom pop-up messages. We addressed this with a Zoom instruction guide tailored for users (Multimedia Appendix 1). It covered how to join the meeting, how to start and stop screen sharing, how to use optional features (share computer sound and optimize for full-screen video), and how to leave the meeting.

#### Step 5: Conduct Testing and Analysis

##### Considerations

Usability scripts guide participants through the required navigation tasks and assess knowledge acquisition. Testing can be conducted using synchronous (moderated in real time) usability testing. Typically, formal testing requires a sample size of 10 to 12 users, and less formal, asynchronous testing requires 4 to 5 users [1,14]. This number is sufficient to uncover approximately 80% of usability issues [1,14]. Beyond this, it is unlikely that more users will elicit new information, with the most severe usability issues consistently detected in that initial small sample of users [1,14]. Subjective assessment of usability can be conducted using the System Usability Scale (SUS), a simple 10-item Likert scale [2,20]. Means and SDs for overall, individual, and subgroup SUS scores can be calculated using the SUS Calculator software package [15]. Other outcomes can include task completion time and rates based on screen recordings, knowledge acquisition through teach back, and user experience feedback via a customized questionnaire.

##### Case Examples

Online and in-person synchronous sessions with survivors of stroke without communication difficulties and caregivers were moderated by author KN. In-person sessions with survivors of stroke with communication difficulties were moderated by author EG (research speech pathologist with expertise in aphasia-friendly practices), with support from KN for task instructions.

Usability testers followed a prepared script to ensure that there was consistency across all sessions and between moderators (Multimedia Appendix 2). The same scripts were used for survivors of stroke with and without communication difficulties, but scripts for people with communication difficulties were delivered in person by EG using aphasia-friendly practices [21]. Strategies included simple sentence structure, slow speaking rate, visual supports, gestures, drawing, and emphasis on keywords. We used “yes” or “no” responses with written support. All sessions (including those conducted in person) were video recorded using the recording function on Zoom for remote sessions and screen recording for in-person sessions. Sessions commenced with introductions, a description of the project, and an outline of how the session would flow. The moderator guided participants through each session by providing testing questions that were either task based or knowledge based and observing participant behavior. Moderators checked in regularly with users to see whether they wanted to take a rest break.

Task-based testing was recorded as a binary outcome of “yes” (completed) or “no” (not completed). Interrater reliability of task-based testing was not assessed given the binary nature and predefined scores (no interpretation by the reviewer was required). Knowledge acquisition was analyzed using the “chunk and check” technique, where one task is given at a time (chunk) and then followed by “teach back” (check), ensuring repetition if needed, checking comprehension, and maximizing recall [22]. Accuracy of responses was coded according to a rating scale of 0 for accurate, 1 for partially accurate, or 2 for inaccurate. Interrater reliability of coding knowledge acquisition was achieved through simple percentage of agreement on codes. Each code had corresponding definitions to guide moderators in categorizing testers’ responses. As shown below, percentage of agreement was calculated by dividing the number of instances in which KN and EG agreed on the code chosen (rater agreement) by the number of items (teach-back tasks) and then multiplied by 100 to obtain an overall percentage of reliability [22,23].

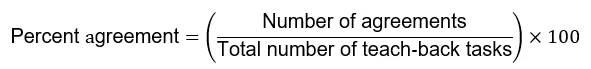



The raw mean SUS score was normalized by establishing a benchmark percentile of the website’s position when compared to other websites (Multimedia Appendix 3). To determine whether differences in SUS scores in the different groups of users were significant, subgroup mean scores for survivors of stroke with communication difficulties were compared to those of survivors without communication difficulties using a between-subject 2-sample 2-tailed *t* test, with statistical significance set at a *P* value of .05 or lower (Multimedia Appendix 4). Data on the user experience survey were tabled against the data on task time and task completions. This allowed the team to examine usability from different perspectives, providing an opportunity to make targeted website changes when issues were identified.

## Discussion

### Principal Findings

This paper describes the development and application of a methodological approach for the usability testing of a novel website to build self-efficacy for survivors of stroke. Our methodological approach was designed to assess how users navigate through the website, whether they could find relevant information, and whether the information was easy to understand. We also evaluated satisfaction with the website to support changes and/or confirm its readiness for release. This work advances the field of digital design in health by promoting explicit reporting of methodological approaches for accessible usability testing.

We specifically sought to include survivors of stroke with and without communication difficulties in our usability testing to ensure that the website was accessible to these groups. However, published evaluation and usability testing processes of health interventions frequently lack clear, detailed descriptions [24]. Additionally, there is no standardized framework for planning these evaluations with people with disabilities, such as survivors of stroke [24]. Involving survivors of stroke with communication difficulties is even more challenging due to the complex language requirements of many evaluation procedures [25]. Given the lack of guidance available on how to conduct usability testing with survivors of stroke with and without communication difficulties, we have developed and described our methods in this tutorial. We anticipate that this tutorial will be of benefit to future designers and researchers who seek to create digital resources for people with neurological conditions, communication impairments, or other barriers to participating in usability testing of a digital resource.

Bringing together experts in the field of digital design, health care, and lived experience was important to facilitate both digital design and usability testing. In this project, we demonstrated that people with great needs, specifically survivors of stroke with communication difficulties, who often experience poorer outcomes and are routinely excluded from research, can be meaningfully included in decision‑making processes. Their active participation shows that they can evaluate whether a digital resource meets their needs and identify where adaptations are required [9,26,27]. We consider that more research needs to be conducted that focuses on rather than excludes people with communication difficulties. Furthermore, it is important to recognize that there are strategies and processes that can enable full participation of people with communication difficulties in research activities [8]. It was imperative that we adapt our usability testing procedures to meet the needs of this group. Our rigorous planning processes, where digital designers provided input about usability testing processes and health professionals and people with lived experience of communication difficulties advised on adaptations required, resulted in a successful usability testing process. Our template can be used and adapted to meet the needs of other underserved yet high-need groups who may face more barriers to both accessing digital resources and participating in usability testing and other forms of research.

### Future Implications

The inclusive framework outlined in this tutorial highlights the importance of prioritizing accessibility from the outset in project planning for digital health intervention developers and researchers. Furthermore, it can be adapted for other populations with neurological or cognitive impairments, such as traumatic brain injury, dementia, or developmental disorders. By demonstrating that survivors of stroke, including those with communication difficulties, can meaningfully participate in usability testing, this approach challenges existing norms and promotes equity in digital health design. Policymakers and funding bodies may be influenced to support inclusive design practices as a standard requirement for digital health design.

### Limitations

We were unable to recruit survivors of stroke or carers with significant vision impairments as the website’s read-aloud functionality was not available during the testing period. In addition, this study did not incorporate a targeted strategy to engage users from culturally or linguistically diverse backgrounds; all participants spoke English as their first language. These limitations highlight challenges in achieving inclusive usability testing and reinforce the importance of adopting more adaptive and accessible research approaches. As a result, the usability outcomes for the EmpowerMe website may have limited generalizability, particularly in relation to populations not represented in the users.

### Conclusions

This tutorial highlights the importance of inclusive formal usability testing in the development of digital health tools. By involving survivors of stroke, including those with communication difficulties, and caregivers, we improved the EmpowerMe website relevance and accessibility, resulting in a replicable framework for others to use. The adapted testing procedures and team collaboration demonstrate that meaningful inclusion is feasible and beneficial.

## Data Availability

The datasets generated or analyzed during this study are available from the corresponding author on reasonable request.

## Appendix

Multimedia Appendix 1 Zoom (Zoom Video Communications) instructions.

Multimedia Appendix 2 Usability testing scripts.

Multimedia Appendix 3 Normalized System Usability Scale scores.

Multimedia Appendix 4 System Usability Scale between-subject score comparison.

## Abbreviations

SUS: System Usability Scale
