# Supplementary material for: Developing and Applying a Usability Testing Methodology for a Website With Survivors of Stroke and Caregivers: Tutorial
Source: J Med Internet Res. 2026 Aug 18;28:e86143. doi: 10.2196/86143 (PMC13535304; doi:10.2196/86143)

Flinders University

Joining a Zoom Meeting:

1. **Check Your Email:** You'll receive an email from the meeting organizer (e.g., Katie Nesbitt) with a link to the Zoom meeting.
2. **Click the Link:** When it's time for the meeting, click the provided link in the email. This will open your web browser.
3. **Ignore the Pop-Up:** If a pop-up window prompts you to download the Zoom application, click "Cancel."
4. **Join from Your Browser:** Look for the "Join from your Browser" link on the screen. Click it.
5. **Set Up Audio:** If you encounter any sound issues, make sure your camera and microphone permissions are enabled.

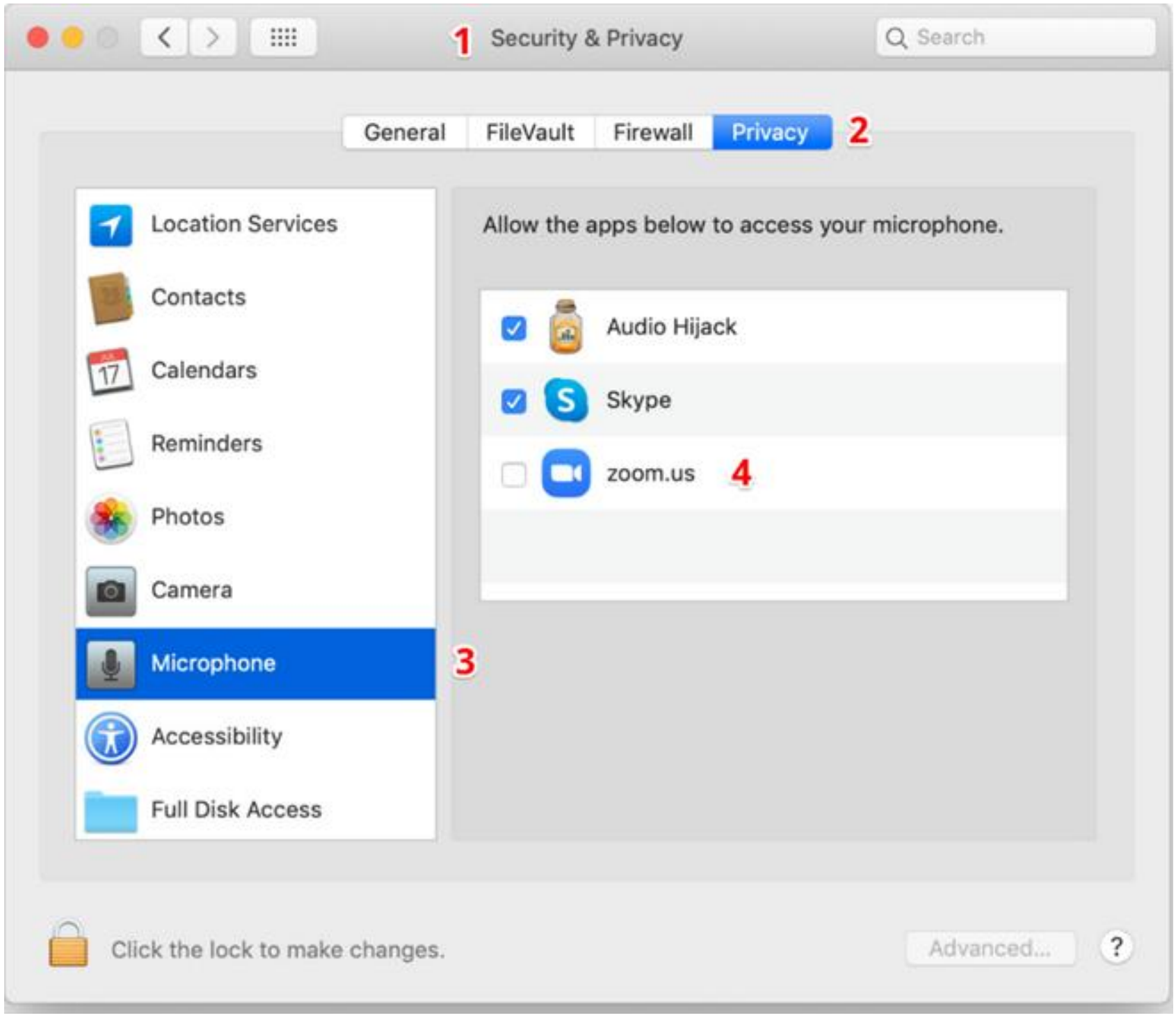

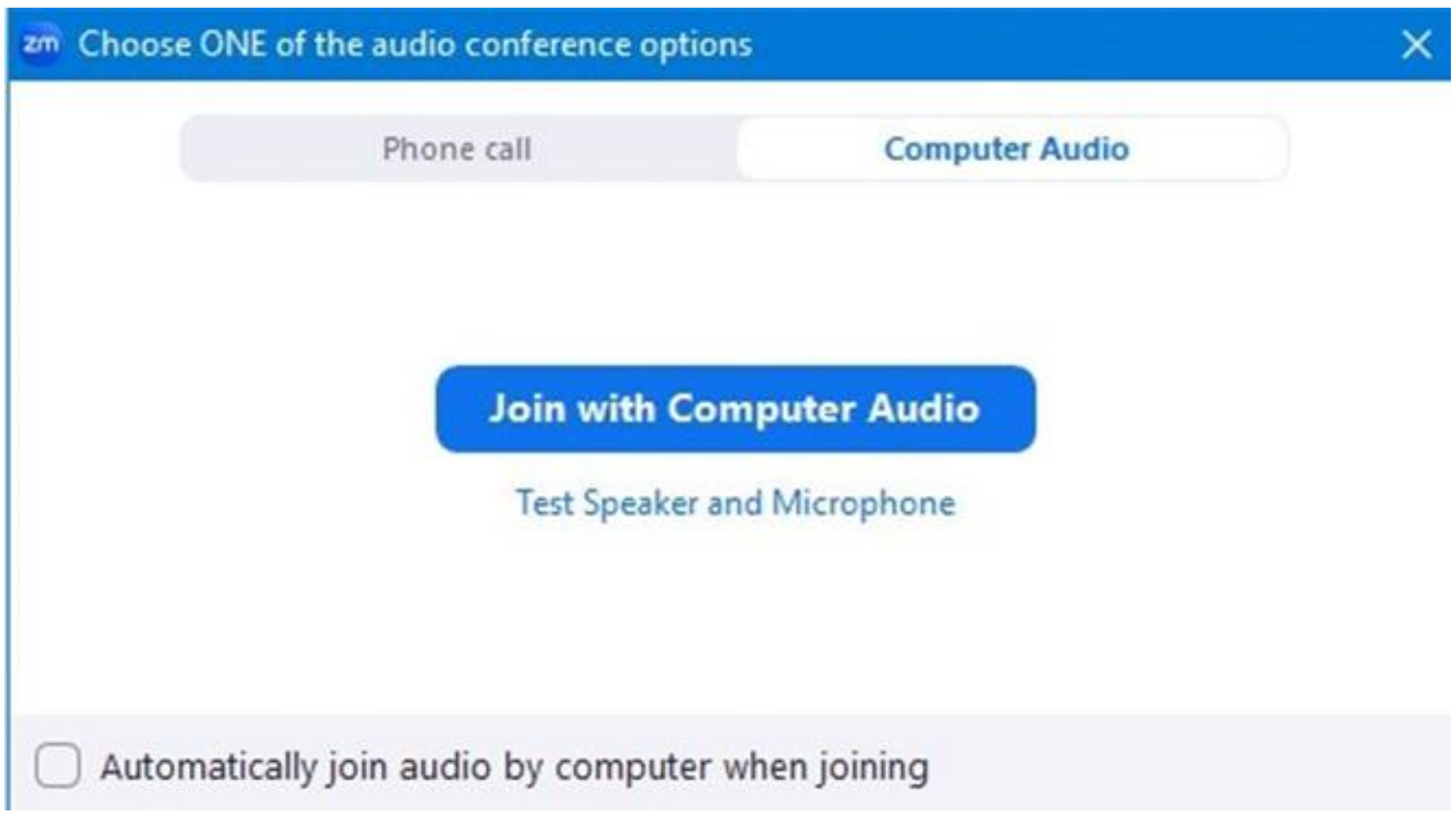

6. **Enter Your Name:** In the "Your Name" field, enter a display name.
7. **Click "Join":** Finally, click the "Join" button to enter the meeting.

Sharing Your Screen:

During the meeting, you will need to share your screen. Follow these steps:

1. **Click "Share Screen":** Locate the "Share Screen" button in the meeting control bar.

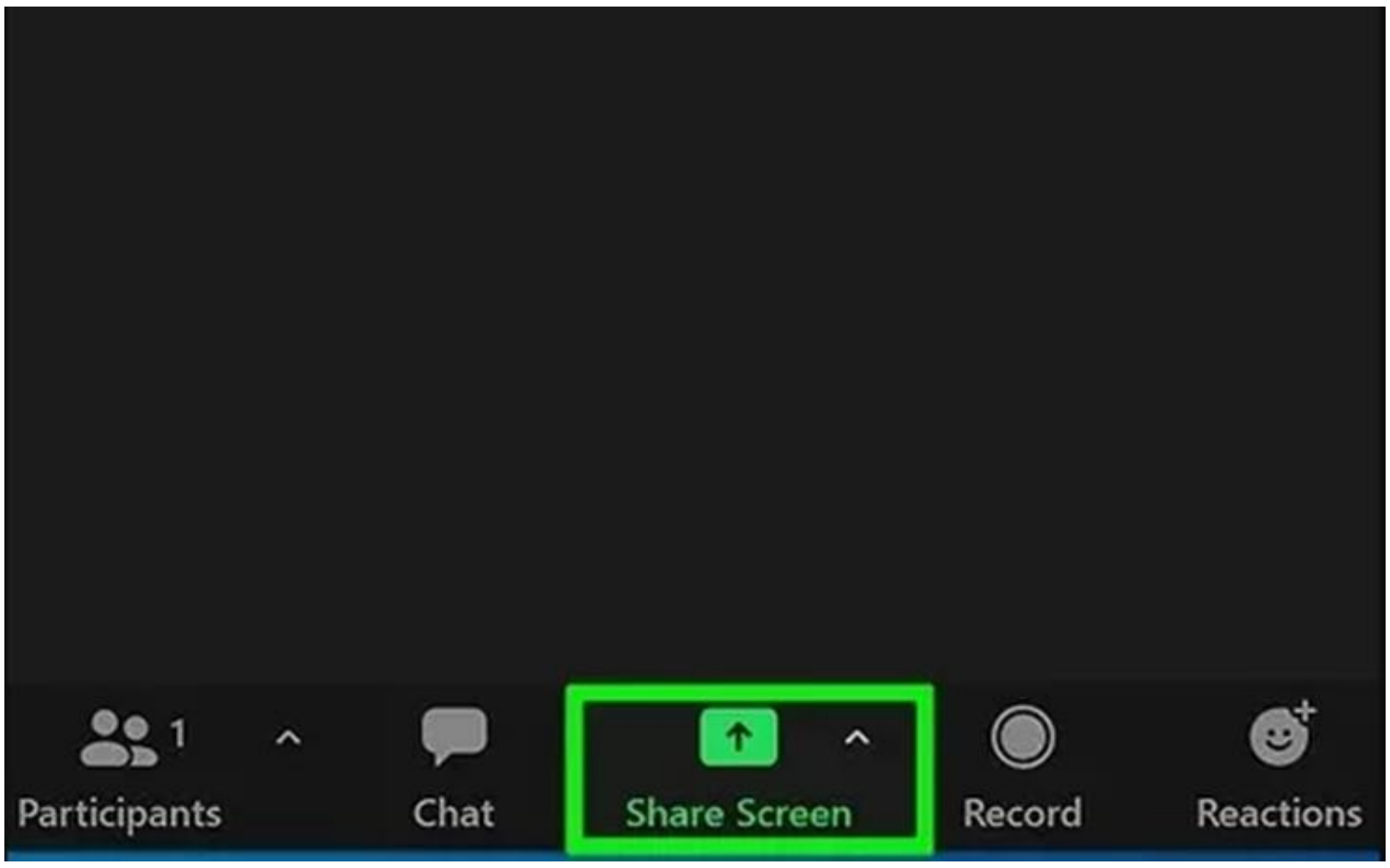

2. **Choose Your Screen:** Select the screen you want to share (e.g., an open application, your desktop, or an iPad screen).

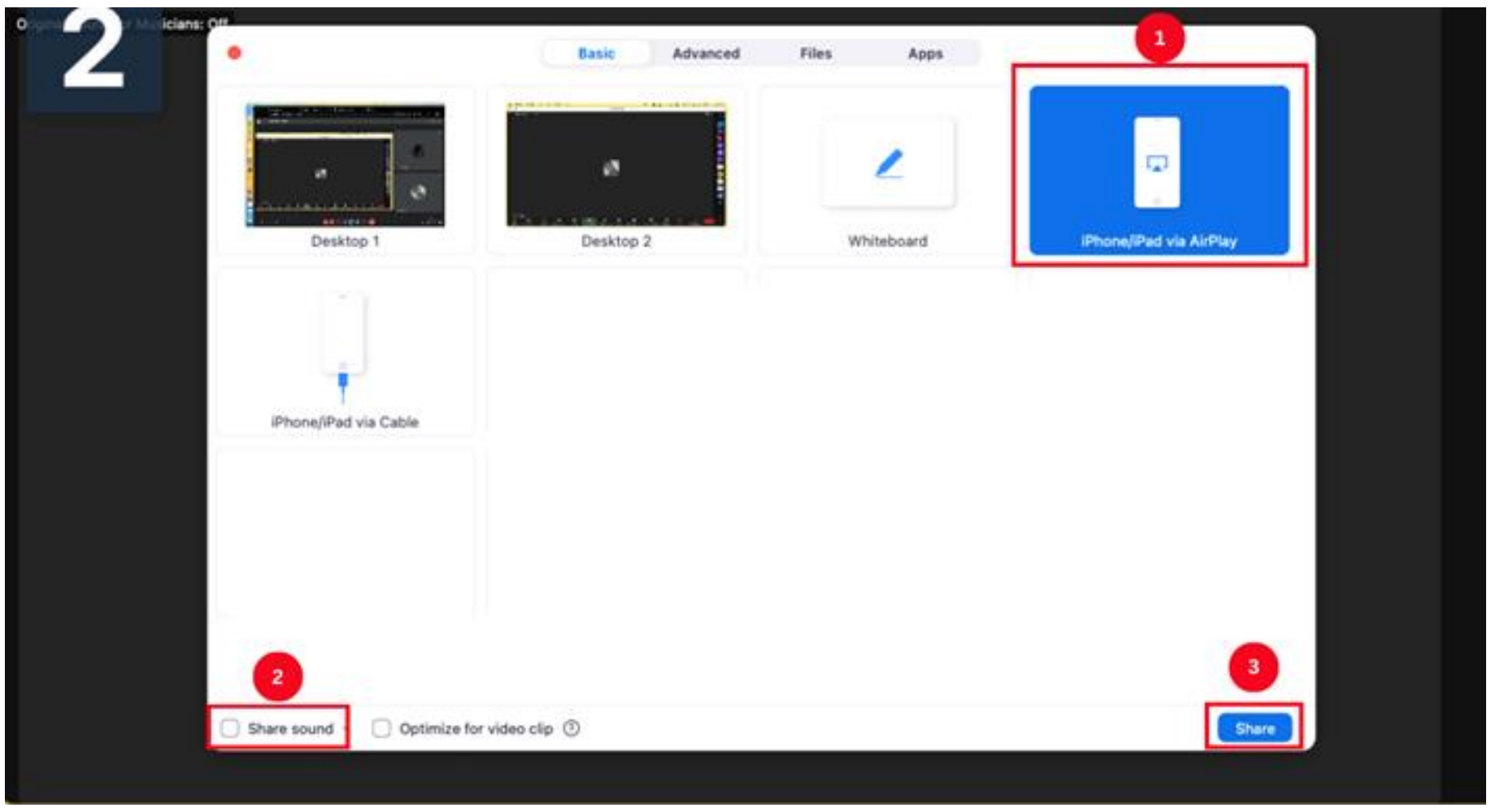

3. **Additional Features (Optional):**
   - **Share Computer Sound:** Enable this option if you want to broadcast your computer's sound to all attendees.

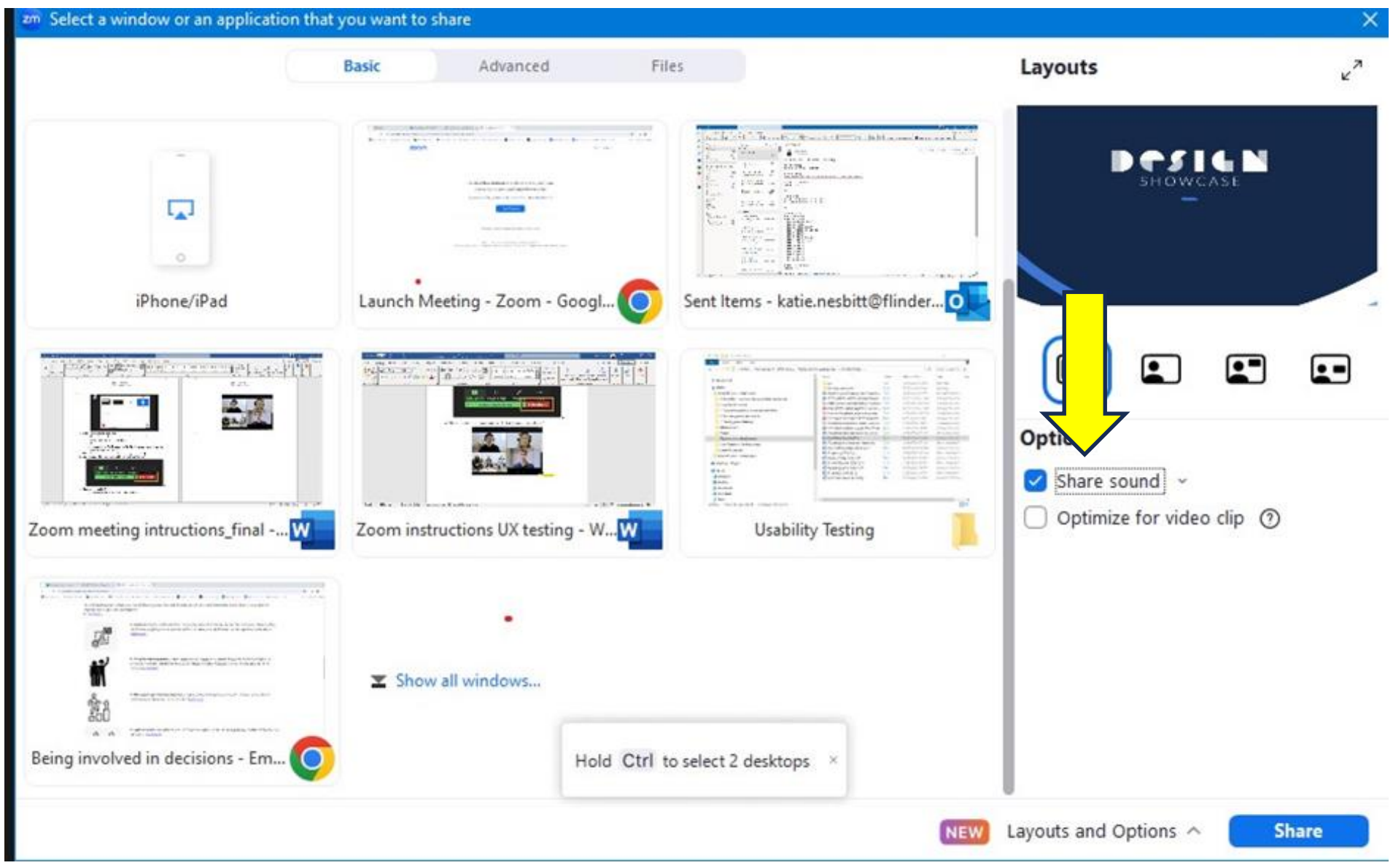

   - **Optimization for Full-Screen Video Clip:** Check this box if you plan to share a video in full-screen mode.
4. **Click "Share":** Share your screen with the meeting participants.
5. **Stop Sharing:** When you're done, click the "Stop Share" button.

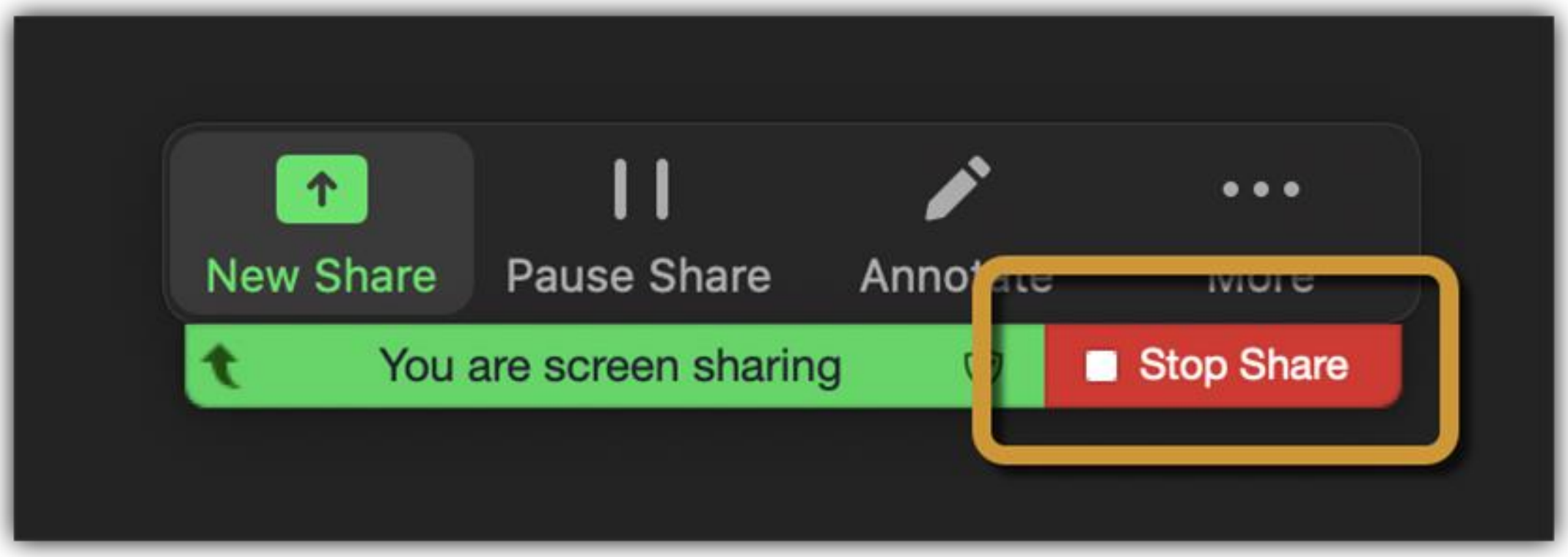

6. **Leaving the Meeting:**
   - To leave the meeting, click the "Leave" button.

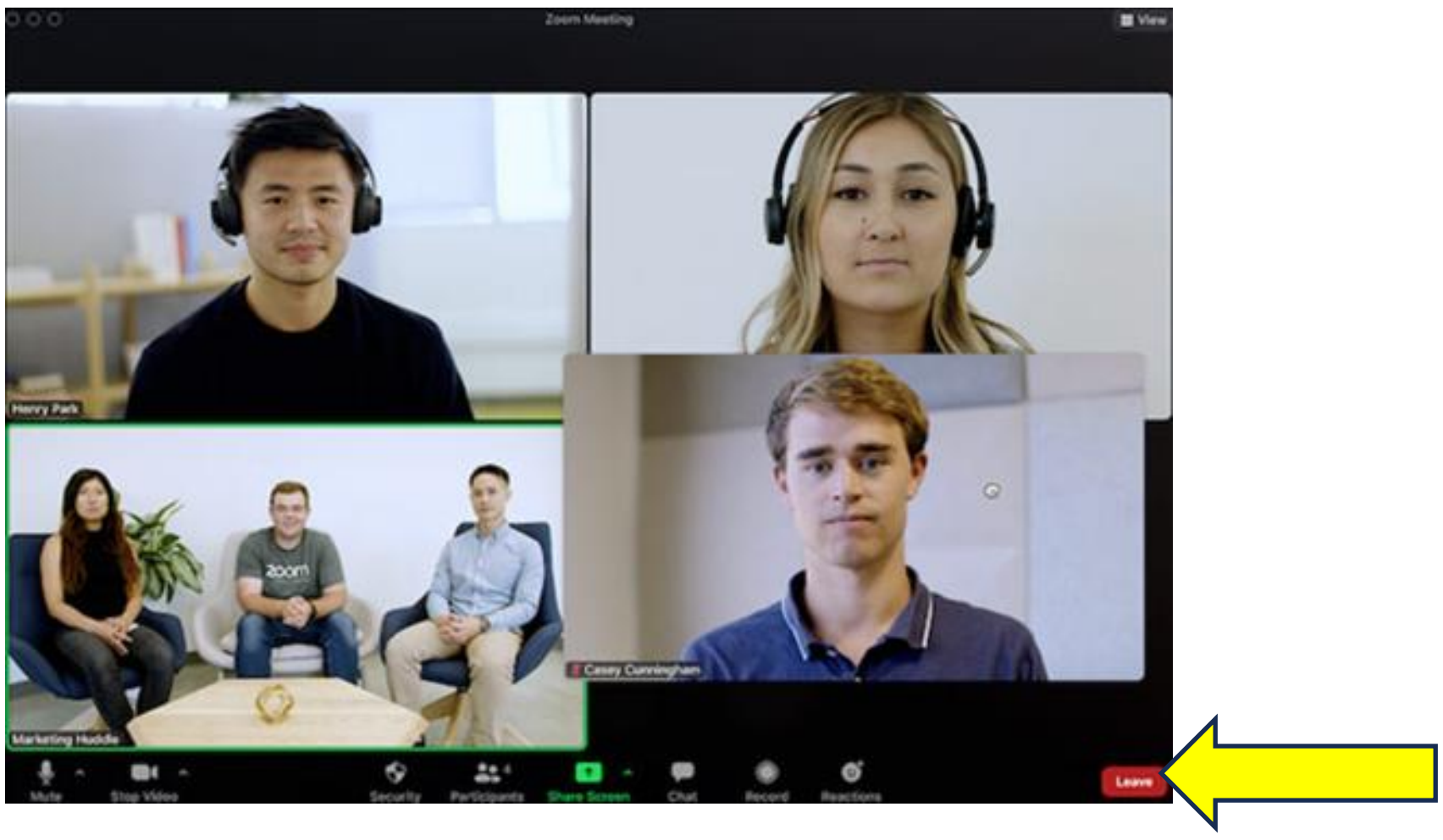

Supplement: Supplementary file 1 [file jmir_v28i1e86143_app1.pdf]
