# Supplementary material for: Developing and Applying a Usability Testing Methodology for a Website With Survivors of Stroke and Caregivers: Tutorial
Source: J Med Internet Res. 2026 Aug 18;28:e86143. doi: 10.2196/86143 (PMC13535304; doi:10.2196/86143)
Supplement: Supplementary file 2 [file jmir_v28i1e86143_app2.docx]

**Appendix 3**

**Usability session script (task)**

| **Hypothesis** | **Carer task** |
| --- | --- |
| Find self-efficacy video on landing page | *Go to information on self-efficacy* |
| Locate and watch video on self-efficacy | *Open, enlarge, watch, reduce video on self-efficacy* |
| Complete resources tailoring tool | *Find resources that would be helpful to you* |
| Open a specific resource | *Read one of the resources* |
| Navigate back to the website | *Read another resource* |
| MOVE TO SCENARIO BASED TASKS | |

**Usability session script (knowledge)**

| **Hypothesis** | **Survivor/carer task** | **Outcome *(facilitators teach back questions)*** |
| --- | --- | --- |
| Find self-efficacy video on landing page | *Go to information on self-efficacy* |  |
| Locate and watch video on self-efficacy | *Open, enlarge, watch, reduce video on self-efficacy* |  |
| **CHUNK AND CHECK** | | "I want to make sure the website explains things clearly and in a way that is interesting. If you will tell me what is self-efficacy?  Why is it important to build this after having a stroke? |
| Complete resources tailoring tool | *Find resources that would be helpful to you* |  |
| Open a specific resource | *Read one of the resources* |  |
| **CHUNK AND CHECK** | | “You have gone over a lot of information, and I want to make sure it is clear to you. So, tell me, what do you think are the three most important things to know about what you have just read? (this will depend on what resource they read) |
| Navigate back to the website | *Watch the video on practice* |  |
| **CHUNK AND CHECK** | | It important to us that this information is clear for people using the website. Can you describe how practicing a task builds self-efficacy? |
| Navigate to the topics, choose one, read/watch/listen   TEST READ ALOUD FUNCTION HERE | *Choose a topic that is interesting or relevant to you*  Imagine you have a sight problem…now listen to the page you are on |  |
| **CHUNK AND CHECK** | | Has this information built your self-efficacy, and if so, can you tell me how? |
