# Supplementary material for: Developing and Applying a Usability Testing Methodology for a Website With Survivors of Stroke and Caregivers: Tutorial
Source: J Med Internet Res. 2026 Aug 18;28:e86143. doi: 10.2196/86143 (PMC13535304; doi:10.2196/86143)
Supplement: Supplementary file 3 [file jmir_v28i1e86143_app3.docx]

**Appendix 5 System Usability Raw score to percentile**

Note: This means that a raw SUS score of 78 is considered more usable that 82.9% of all products tested using the SUS. Anything below 50% is below average and anything above 50% is above average. Calculations: *Actual Benchmark 67.000, Reflected Score 22.0, Reflected Benchmark 33, Ln Reflected Score	3.09, Ln (logarithmus naturali), Reflected Benchmark 3.497, Population SD13.400, Ln (logarithmus naturali) SD 0.426727, z	-0.950, Reflected % 0.171, % 0.829*
