# Supplementary material for: Developing and Applying a Usability Testing Methodology for a Website With Survivors of Stroke and Caregivers: Tutorial
Source: J Med Internet Res. 2026 Aug 18;28:e86143. doi: 10.2196/86143 (PMC13535304; doi:10.2196/86143)
Supplement: Supplementary file 4 [file jmir_v28i1e86143_app4.docx]

Appendix 6-System Usability Scale between-subjects comparison

|  |  |
| --- | --- |
| Survivors of stroke without communication difficulties | 86.875 |
| Survivors of stroke with communication difficulties | 61.25 |

Note: without communication difficulties, n=4, mean 86.9 (SD 2.4), with communication difficulties, n=4, mean 61.2 (SD 6.6).

Note: Confidence interval -36.8, -14.4, observed difference 25.6.
